# Case report: Hybrid approach in the management of a complete persistent sciatic artery aneurysm

**DOI:** 10.1016/j.ijscr.2020.11.077

**Published:** 2020-11-19

**Authors:** Andrew Yang, Ammar Hashmi, Anton Androsov, Gregory Salzler, Evan Ryer

**Affiliations:** Department of Endovascular & Vascular Surgery, Geisinger Medical Center, Danville, PA, United States

**Keywords:** Persistent sciatic artery, Aneurysm, Endovascular, Hybrid, Bypass

## Abstract

**Introduction:**

Persistent sciatic artery (PSA) is a rare embryological vascular anomaly with a prevalence between 0.025–0.06%. PSA is frequently associated with aneurysmal degeneration and can result in neuropathy, thrombosis, or rupture, threatening limb and life.

**Case presentation:**

We present a case of a 72-year-old man with an incidental finding of a right sided 4 cm PSA aneurysm with limited symptoms. The aneurysm was treated successfully with endovascular exclusion and a femoral-popliteal bypass was performed to revascularize the leg.

**Discussion:**

Treatment of PSA aneurysms involve excluding the aneurysm and revascularizing the involved leg. Improvements in endovascular embolization techniques now offer new solutions in the management of these aneurysms.

**Conclusion:**

A high degree of clinical suspicion is required to properly diagnose and treat PSA aneurysms. Referral to a center with expertise in both open and endovascular techniques is vital to ensure good outcomes.

## Highlights

• Persistent sciatic arteries are an uncommon embryological vascular phenomenon.
• Persistent sciatic arteries are usually asymptomatic but may become aneurysmal.
• Aneurysmal degeneration can cause neuropathy and embolic complications.
• Both open surgical and endovascular approaches may be suitable treatment options.

## 1 Introduction

A persistent sciatic artery (PSA) is an uncommon embryological vascular phenomenon [1]. The first description of PSA was outlined by PH Green in *The Lancet* in 1832 with the first mortality due to a ruptured aneurysm noted in 1864 [1,2]. The incidence of PSA in the current literature is between 0.025–0.06% [1,3]. In most cases, PSA presents unilaterally, with the right side being more predominant. Bilateral PSAs account for up to 30% of all cases. Both sexes are affected equally [3]. The presence of PSA is typically asymptomatic, however, aneurysmal formation has been noted in 14.3–44% of all cases [1,4]. Most patients diagnosed with PSA complications are between ages forty and fifty and present with painful pulsatile buttock masses, lower limb ischemia, or neuropathy due to nerve compression [3,4]. Familiarity with this congenital condition is crucial in order to avoid further complications of lower limb ischemia and inappropriate interventions in cases of misdiagnosis of arterial occlusion. We are presenting a case of a 72-year-old man with an incidental finding of a right sided 4 cm PSA aneurysm. This case is presented in accordance with the SCARE 2018 guidelines [5].

## 2 Case report

**Fig. 1:**
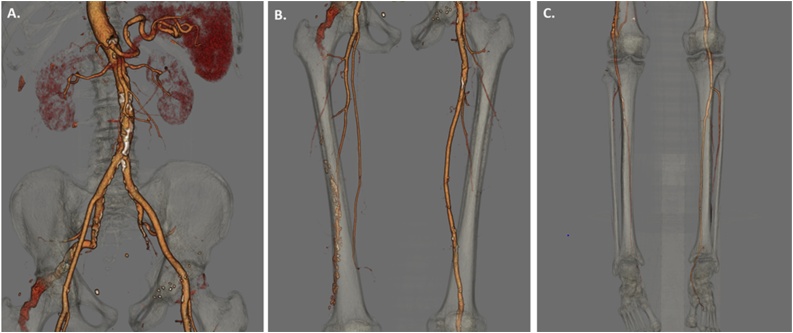
Computed tomography (CT) reconstructions demonstrating a right-sided persistent sciatic artery aneurysm. A) The right PSA is a continuation of the internal iliac artery and exits the pelvis caudally. B) The right PSA courses inferiorly along the entire right thigh. C) Continuation of right PSA as a popliteal artery. Note the hypoplastic nature of the distal right superficial femoral artery.

**Fig. 2:**
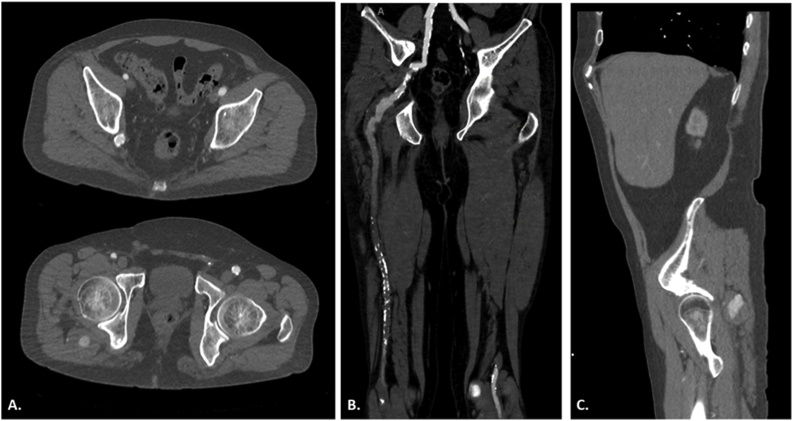
A) Computed tomography (CT) axial images at the gluteal region demonstrating a right-sided persistent sciatic artery (PSA) aneurysm. B) Coronal view of the posterior thigh elucidating the path of the right PSA from iliac artery to popliteal artery. C) Sagittal view confirming the size and location of the right PSA aneurysm.

A 72-year-old man with a past medical history of hypertension, diabetes mellitus, coronary artery disease, and aortic insufficiency was admitted to our hospital with an unprovoked pulmonary embolus. As part of an occult malignancy work-up, a computed tomography (CT) of the abdomen and pelvis with intravenous (IV) contrast was performed and revealed a right persistent sciatic artery aneurysm. Follow-up CT angiography confirmed the diagnosis of a right sided persistent sciatic artery aneurysm and also demonstrated a hypoplastic right superficial femoral artery (SFA). (Fig. 1). Maximal aneurysm dimensions were 40 mm × 35 mm in the right gluteal region (Fig. 2). The patient did not endorse any right lower extremity complaints. Physical exam was notable for a pulsatile right sided gluteal mass, bilateral palpable femoral pulses but no palpable pedal pulses.

**Fig. 3:**
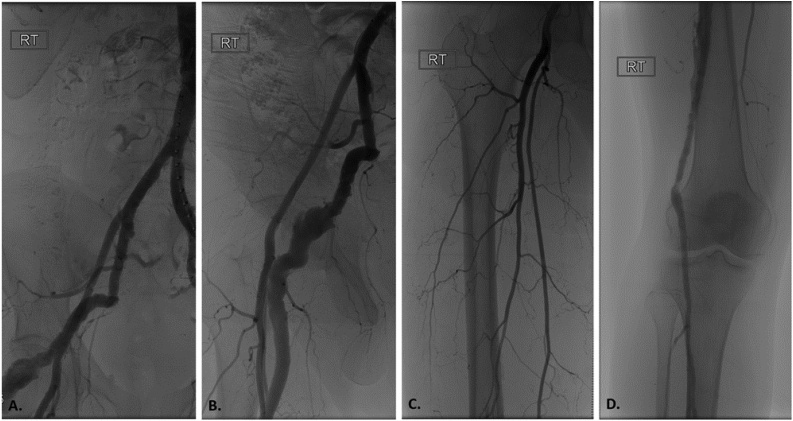
Intra-operative angiography scan prior to persistent sciatic artery (PSA) aneurysm embolization and femoropopliteal bypass. A, B, D) Angiography demonstrating a complete type PSA along with a hypoplastic superficial femoral artery (C).

**Fig. 4:**
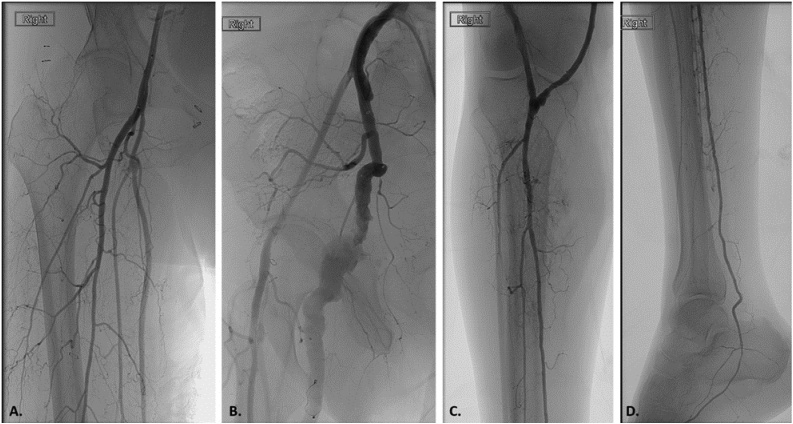
Intra-operative angiography following persistent sciatic artery (PSA) aneurysm embolization and femoropopliteal bypass. A, C) Completion angiography of the proximal superficial femoral to below knee popliteal artery saphenous vein bypass. B) Proximal and distal embolization of the PSA aneurysm. D) Completion angiography demonstrating single vessel runoff via a patent right posterior tibial artery.

After a standard pre-operative evaluation, the patient elected to proceed with surgery to reduce the potential risk of aneurysm rupture, thrombosis or embolization (Fig. 3). From a percutaneous left femoral access, two 16 mm Amplatzer ™ vascular plugs (Abbott Medical, Plymouth, MN) were placed proximally and distally to exclude the PSA aneurysm. Next, standard exposure of the proximal superficial femoral and below knee popliteal arteries was performed. Ipsilateral saphenous vein was harvested and a non-reversed proximal superficial femoral to below knee popliteal artery bypass was performed to restore perfusion to the patients right lower extremity (Fig. 4). The proximal superficial femoral artery was chosen as inflow for the bypass due to limited saphenous vein conduit length. His surgery was performed by one of our fellowship trained vascular surgeons at a facility with an endovascular capable operating suite. After an uneventful three-day hospital stay, the patient was discharged in a stable condition. Aside from the immediate post-operative recovery, he has had no changes to his level of activity or lifestyle.

A CT angiogram of the aorta with lower extremity run off was obtained one month following his embolization which demonstrated a successfully excluded right sciatic artery. There was no flow appreciated within the aneurysm sac. His post-operative arterial doppler study demonstrated an ankle brachial index of 1.0 with triphasic wave forms. He continues to follow with surveillance arterial duplex studies obtained every 6 months. His graft has remained patent at his one year follow up with no symptoms.

## 3 Discussion

A persistent sciatic artery (PSA) is a developmental anomaly with an unusually high incidence of complications [6]. In the first trimester of embryological development, the sciatic artery serves as the major blood supply to the lower limb bud [7]. After the third month of development, the sciatic artery regresses to form the proximal part of the inferior gluteal artery while the femoral artery continues to develop and provides the major blood supply to the lower extremity. Failure of involution results in either a complete or incomplete type of PSA [2,3]. The most common manifestation, the complete type PSA, continues into the popliteal artery and serves as the main blood supply for the lower extremity. The superficial femoral artery is frequently hypoplastic with a complete type PSA. An incomplete PSA is frequently hypoplastic with the superficial femoral artery as the main supply for the lower limb [2]. Regardless of the type, a PSA is often tortuous and diffusely enlarged [7].

Aneurysmal degeneration of persistent sciatic arteries has been noted in 14.3–44% of all cases [1,6]. Most patients present with unilateral and complete type PSAs. The typical symptoms include a painful pulsatile buttock mass, sciatic neuropathy, or lower limb ischemia due to thromboembolization [4]. Classic physical exam findings include an absent femoral pulse with palpable distal pulses. Asymptomatic cases are rare and incidental [3]. The high incidence of aneurysmal degeneration can be explained by a congenital lack of elastic tissue and the vulnerable anatomic position of the artery [2]. The anatomy of a PSA subjects it to repetitive trauma through extended periods of sitting, which contributes to the formation of PSA aneurysms [8]. If a patient presents with pulsatile buttock mass, duplex ultrasound can confirm the suspicion of PSA. Arteriography, CT angiogram, or magnetic resonance imaging can elucidate PSA type and provide more diagnostic information [9].

Treatment for PSA includes exclusion of any aneurysms and revascularization of lower extremity if the PSA serves as the limbs dominant blood supply [10]. Complete type PSAs usually require aneurysm embolization and revascularization of distal limb with femoral-popliteal bypass, while aneurysmal exclusion only is appropriate for incomplete type PSA [10,11]. In recent years, endovascular embolization techniques have been developed to reduce the risk of complications, such as sciatic nerve injury.^10^ Our case is a demonstration that endovascular embolization can provide adequate exclusion of the aneurysm without subjecting the patient to an extensive dissection of the aneurysm.

Due to its rare occurrence and high complication potential, a high degree of clinical suspicion is required to properly diagnose and treat PSA aneurysms. Referral to a center with expertise in both open and endovascular techniques is vital to ensure good outcomes.

## Declaration of Competing Interest

The authors report no declarations of interest.

## Sources of funding

No funding was received for this case report.

## Ethical approval

This case report was exempt from ethical approval by our institution policy. Informed consent was obtained to publish this case prior to drafting this manuscript.

## Consent

Written informed consent was obtained from the patient for publication of this case report and accompanying images. A copy of the written consent is available for review by the Editor-in-Chief of this journal upon request.

## Author contribution

Andrew Yang MD, Ammar Hashmi MD, Anton Androsov BS were all involved in writing the paper.

Gregory Salzler MD and Evan Ryer were involved in the care of this patient and assisted with reviewing and editing of this case report.

## Registration of research studies

This is a case report that is not a “first in man” study. The techniques and medical equipment used have been proven to be safe for use in humans for similar disease processes.

## Guarantor

Andrew Yang MD.

## Provenance and peer review

Not commissioned, externally peer-reviewed.
